# A Case of Relapsing Polyarthritis Associated With Hidradenitis Suppurativa: Case Report and Review of Literature

**DOI:** 10.1177/2324709616677064

**Published:** 2016-11-17

**Authors:** Avinash Adiga, John Pixley

**Affiliations:** 1 Texas Tech University Health Sciences Center, Lubbock, TX, USA

**Keywords:** hidradenitis suppurativa, relapsing polyarthritis, spondyloarthritis, musculoskeletal manifestation, HLA-B27 negative

## Abstract

Hidradenitis suppurativa (HS) is a chronic, inflammatory follicular skin disease with recurrent skin nodules, sinus tracts, and scarring. We observed a case of HS associated with relapsing polyarthritis. On presentation the patient had a flare of polyarthritis with an increase in the number and size of pustular nodules. He has had similar episodes 1 to 2 times yearly subsiding with antibiotic treatment. Radiographs revealed erosions and demineralization. Symptoms improved following institution of anti-inflammatory and antibiotic therapy. HS is associated with several inflammatory conditions, and dysregulation in innate immunity may play an important role in etiopathogenesis. Spondyloarthritis/sacroiliitis is the most common joint manifestation in HS and mechanism(s) underlying arthropathy is unknown. Treatment of arthritis in HS is anecdotal.

## Introduction

Hidradenitis suppurativa (HS) is a chronic disorder involving apocrine glands characterized by comedo-like follicular occlusion and chronic relapsing skin inflammation; sinus tracts and scarring may be seen in later stages.^1^ We observed a case of HS in a young African American male with relapsing polyarthritis. Polyarthritis in our patient was associated with flare of the skin lesions and would resolve with anti-inflammatory and antibiotic therapy.

## Case Report

**Figure 1.**
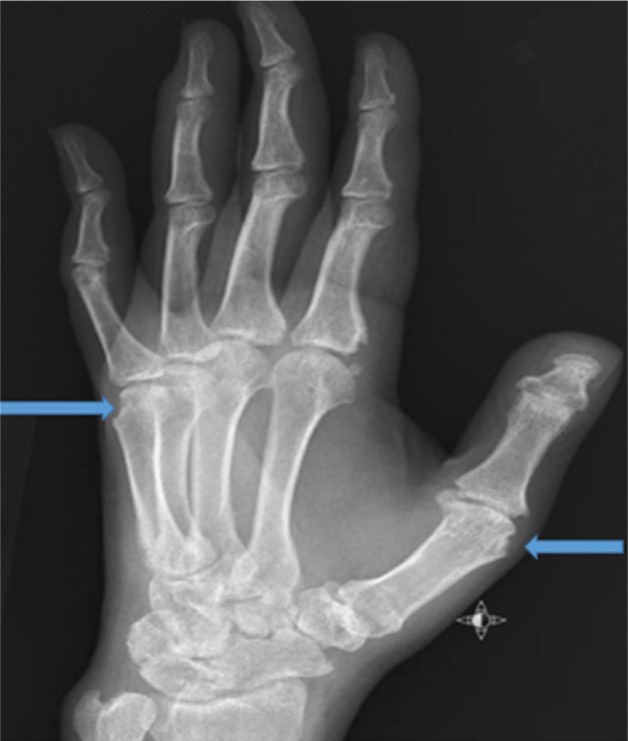
Hand X-ray showing erosions.

**Figure 2.**
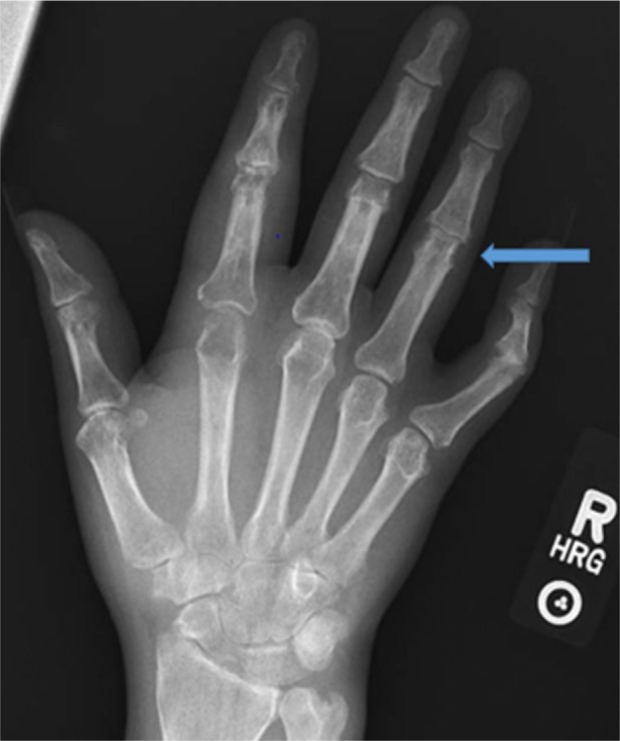
Hand X-ray suggesting early pencil cup deformity.

**Figure 3.**
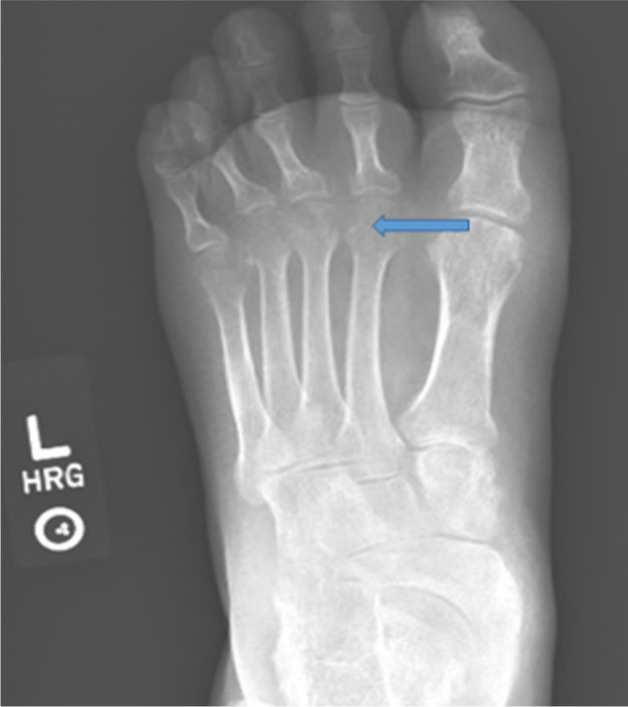
Foot X-ray showing metatarsal erosions.

**Figure 4.**
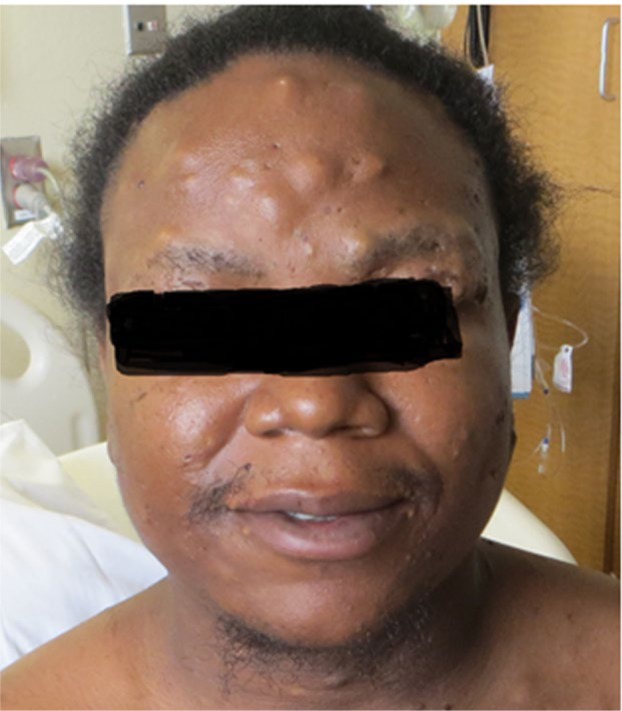
Nodular lesions on forehead.

A 33-year-old African American male with HS (onset as a teenager), with subsequent arthritis 10 years later, presented with multiple nodular skin lesions, predominantly in intertriginous areas in the axilla and inguinal area. He reported an increase in the number and size of draining pustules. He reported generalized joint pains including the left knee and right elbow with swelling and redness. He reports these flares typically occur 1 to 2 times yearly, subsiding in association with antibiotic treatment. Skin examination revealed generalized suppurate nodules and chronic scars (see figure 4). Sinus tracts were noted in the intertriginous areas. Polyarthritis with effusions in ankles, knees, elbows, wrists, and digits with suggestion of a sausage digit right second phalanx was noted on joint exam. Synovial fluid analysis from the left knee revealed yellow cloudy fluid, 190 white blood cells/mm^3^, and 4180 red blood cells/mm^3^, with no crystals seen. Labs revealed leukocytosis (20 000 white blood cells/mm^3^ with neutrophil predominance and no band forms), hemoglobin 12.8 g/dL (normal 13.3-17.1 g/dL), erythrocyte sedimentation rate was 110 mm/h (normal 1-30 mm/hr), high sensitive C-reactive protein 103.2 mg/L(normal 0.1-10 mg/dl). Anti-nuclear antibody, rheumatoid factor, and anti-cyclic citrulline peptide were not detected. Blood and urine culture were negative. Urine *Chlamydia* and *Neisseria* polymerase chain reaction was also negative. Radiographic studies of left hand revealed erosions at the head of second, fourth, and fifth proximal phalanges as well as base of the second middle and proximal phalanges (see Figures 1 and 2). Erosive arthritis was also noted in radiographs of wrist and ankle (see Figure 3). He was empirically started on ceftriaxone and vancomycin for suppurative skin lesions and prednisone for the polyarthritis. His joint pains significantly improved and skin lesions reduced in size and number. On day 5 of admission he was discharged home on sulfasalazine 500 mg daily.

## Background and Discussion

Hidradenitis suppurativa is a chronic, inflammatory, debilitating, painful follicular skin disease presenting as multiple recurrent skin nodules and sinus tracts with subsequent scarring.^1^ It involves the apocrine gland bearing areas most commonly axillary, inguinal, mammary, and anorectal regions. These nodules progresses from noninflamed to inflamed nodules, and may form abscesses that can rupture leading to suppuration and severe malodor. The prevalence of HS is reported to be 1% to 4%,^2,3^ with the age of onset usually between puberty and 40 years of age, occurring 3 times more commonly in women than in men,^4^ with an increased frequency in African Americans. The diagnosis of HS is usually established clinically based on anatomic distribution and morphology. Skin biopsy, bacterial culture, and imaging studies if done are usually performed to rule out other diagnosis.

It has been proposed that hormonal and various dietary factors, notably dairy products, play a role, wherein the follicular unit of the hair follicle gets plugged and distended by keratin. This occlusion results in accumulation of cellular debris and cyst formation. Friction, shearing forces, and pressure leads to rupture of hair follicle, which is followed by a massive local immune response, resulting in painful inflammation, abscess formation, and, in later stages, sinus tract formation and scarring.^5^ The presence of tumor necrosis factor-α (TNF-α) at the mRNA and protein levels has been described in HS skin. Moreover, reports of clinical improvement with infliximab and adalimumab supports the role for TNF-α in pathogenesis.^6,7^ Elevated levels of interleukin (IL)-10, IL-17, and IL-1β have also been noted in studies.^5^ The role of antimicrobial protein in initiation or propagation of HS still remains controversial. Obesity can exacerbate HS by increasing skin-skin and skin-to-cloth friction.^8^

Hidradenitis suppurativa is also associated with metabolic syndrome (obesity, hypertension, diabetes mellitus, and dyslipidemia)^9^ and with several inflammatory conditions: severe acne conglobate, neutrophilic dermatoses, and extracutaneous diseases such as inflammatory bowel disease.^10-13^ Cases of ulcerative keratitis with Moorens ulcer has been described in the literature.^14^

Defective Notch^*^ signaling has been invoked as responsible for both skin and immune manifestations of HS. For example, while it has a significant role in normal follicle development, it can promote epidermal hyperplasia, cyst formation, and a pro-inflammatory environment.^15^ Notch is also an important mediator of T-cell–related immune responses, which can suppress Toll-like receptor-4-induced pro-inflammatory cytokine responses and serve as feedback inhibitor for innate immunity.^16^ Mutation in ψ-secretase (which mediates the intramembranous cleavage of Notch protein) is seen in association with familial HS.^15^ All these conditions are associated with dysregulation of the innate immune system, evidenced by the enhanced presence or expression in tissue lesions of neutrophils and macrophages as well as cytokines, such as TNF-α, IL-1β, and IL-6 .^17^

Musculoskeletal association with HS has been reported. Most note an association with skin flares and that it tends to occur years after the onset of skin disease. Most reports are anecdotal. We identified one multicenter analysis.^18^ The prevalence of spondyloarthopathy in HS patients was found to be 3.7% in this study. Arthritis was usually seronegative, insidious in onset, occurring 2 to 15 years after the appearance of skin lesions. It usually starts as an oligoarthritis, but may progress to asymmetric polyarthritis. Spondyloarthritis/sacroiliitis is the most common joint manifestation noted.^19^ Joints affected includes knees followed by elbow, wrist, ankle, and shoulder. Radiographic findings include soft tissue swelling, periarticular osteoporosis, joint space narrowing, erosions, and periosteal new bone formation.^20^ The arthritis can persist after a flare. Laboratory evaluation in these cases may reveal mild anemia, leukocytosis, and elevated erythrocyte sediment rate. ANA positivity has been reported. HLA-B27 is usually negative. HS with arthritis is also reported in association with pyoderma gangrenosum, recurrent urethritis, conjunctivitis, and xerophthalmia.^19^ An entity called PAPASH syndrome pyogenic (inflammatory) arthritis, pyoderma gangrenosum, acne, hidradenitis suppurativa has been discussed in the literature.^21^

While the mechanism(s) underlying the arthopathy are not known, hypersensitivity to bacterial antigens has been suggested based on the temporal association with the skin flares.^14^ PAPASH has been associated with a mutation of PSTPIP1 gene, but no clear genetic predisposition has been described in HS. No specific bacterium has been identified. Smoking has been suggested as a common triggering factor in both HS and spondyloarthropathies. While the above suggests the arthritis is reactive to skin stimuli, reports of arthritis preceding the skin lesions challenges this proposition.^20^ Treatment of arthritis in HS is anecdotal. Nonsteroidal anti-inflammatory drugs along with prednisone and also disease-modifying antirheumatic drugs such as sulfasalazine and methotrexate have been tried.^20,22^ Infliximab and adalimumab has been effectively used in treatment of spondyloarthropathy in association with HS.^23^

## Conclusion

Inflammatory arthropathy can be a complication of hidradenitis suppurativa.

## Footnotes

* Notch is a type 1 membrane protein involved in intracellular signaling and plays a key role in cell differentiation and tissue homeostasis in multiple adult organ systems including hematopoietic and immune system.

## References

[1] Jemec GB Clinical practice. Hidradenitis suppurativa. N Engl J Med. 2012;366:158-164. 22236226 10.1056/NEJMcp1014163

[2] Jemec GB Heidenheim M Nielsen NH The prevalence of hidradenitis suppurativa and its potential precursor lesions. J Am Acad Dermatol. 1996;35(2 pt 1):191-194. 8708018 10.1016/s0190-9622(96)90321-7

[3] Shahi V Alikhan A Vazquez BG Weaver AL Davis MD Prevalence of hidradenitis suppurativa: a population-based study in Olmsted County, Minnesota. Dermatology. 2014;229:154-158. 25228133 10.1159/000363381 PMC4216603

[4] Canoui-Poitrine F Le Thuaut A Revuz JE Identification of three hidradenitis suppurativa phenotypes: latent class analysis of a cross-sectional study. J Invest Dermatol. 2013;133:1506-1511. 23235532 10.1038/jid.2012.472

[5] Prens E Deckers I Pathophysiology of hidradenitis suppurativa: an update. J Am Acad Dermatol. 2015;73(5 suppl 1):S8-S11. 26470623 10.1016/j.jaad.2015.07.045

[6] Grant A Gonzalez T Montgomery MO Cardenas V Kerdel FA Infliximab therapy for patients with moderate to severe hidradenitis suppurativa: a randomized, double-blind, placebo-controlled crossover trial. J Am Acad Dermatol. 2010;62:205-217. 20115947 10.1016/j.jaad.2009.06.050

[7] Miller I Lynggaard CD Lophaven S Zachariae C Dufour DN Jemec GB A double-blind placebo-controlled randomized trial of adalimumab in the treatment of hidradenitis suppurativa. Br J Dermatol. 2011;165:391-398. 21457202 10.1111/j.1365-2133.2011.10339.x

[8] Alikhan A Lynch PJ Eisen DB Hidradenitis suppurativa: a comprehensive review. J Am Acad Dermatol. 2009;60:539-561. 19293006 10.1016/j.jaad.2008.11.911

[9] Miller IM Ellervik C Vinding GR Association of metabolic syndrome and hidradenitis suppurativa. JAMA Dermatol. 2014;150:1273-1280. 25229996 10.1001/jamadermatol.2014.1165

[10] Marzano AV Trevisan V Gattorno M Ceccherini I De Simone C Crosti C Pyogenic arthritis, pyoderma gangrenosum, acne, and hidradenitis suppurativa (PAPASH): a new autoinflammatory syndrome associated with a novel mutation of the PSTPIP1 gene. JAMA Dermatol. 2013;149:762-764. 23571383 10.1001/jamadermatol.2013.2907

[11] Braun-Falco M Kovnerystyy O Lohse P Ruzicka T Pyoderma gangrenosum, acne, and suppurative hidradenitis (PASH)—a new autoinflammatory syndrome distinct from PAPA syndrome. J Am Acad Dermatol. 2012;66:409-415. 21745697 10.1016/j.jaad.2010.12.025

[12] Marzano AV Menicanti C Crosti C Trevisan V Neutrophilic dermatoses and inflammatory bowel diseases. G Ital Dermatol Venereol. 2013;148:185-196. 23588144

[13] Van der Zee HH van der Woude CJ Florencia EF Prens EP Hidradenitis suppurativa and inflammatory bowel disease: are they associated? Results of a pilot study. Br J Dermatol. 2010;162:195-197. 19681876 10.1111/j.1365-2133.2009.09430.x

[14] Soukiasian S Foster S Diagnostic Problems in Clinical Ophthalmology. Philadelphia, PA: Saunders; 1994:220-226.

[15] Wang B Yang W Wen W Gamma-secretase gene mutations in familial acne inversa. Science. 2010;330:1065. 20929727 10.1126/science.1196284

[16] Radtke F Fasnacht N Macdonald HR Notch signaling in the immune system. Immunity. 2010;32:14-27. 20152168 10.1016/j.immuni.2010.01.004

[17] Roux CH Saraux A Le Bihan E Rheumatoid arthritis and spondyloarthropathies: geographical variations in prevalence in France. J Rheumatol. 2007;34:117-122. 17117490

[18] Richette P Molto A Viguier M Hidradenitis suppurativa associated with spondyloarthritis—results from a multicenter national prospective study. J Rheumatol. 2014;41:490-494. 24429166 10.3899/jrheum.130977

[19] Rosner IA Richter DE Huettner TL Kuffner GH Wisnieski JJ Burg CG Spondyloarthropathy associated with hidradenitis suppurativa and acne conglobate. Ann Intern Med. 1982;97:520-525. 6214980 10.7326/0003-4819-97-4-520

[20] Bhalla R Sequeria W Arthritis associated with hidradenitis suppurativa. Ann Rheum Dis. 1994;53:64-77. 8311560 10.1136/ard.53.1.64 PMC1005247

[21] Saraceno R Babino G Chiricozzi A Zangrilli A Chimenti S PsAPASH: a new syndrome associated with hidradenitis suppurativa with response to tumor necrosis factor inhibition. J Am Acad Dermatol. 2015;72:e42-e44. 25497954 10.1016/j.jaad.2014.10.002

[22] Thein M Hogarth MB Acland K Seronegative arthritis associated with the follicular occlusion triad. Clin Exp Dermatol. 2004;29:550-552. 15347350 10.1111/j.1365-2230.2004.01582.x

[23] Scheinfeld N Treatment of coincident seronegative arthritis and hidradenitis suppurativa with adalimumab. J Am Acad Dermatol. 2006;55:163-164. 16781316 10.1016/j.jaad.2006.01.024

